# Supplementary material for: DMRforPairs: identifying Differentially Methylated Regions between unique samples using array based methylation profiles
Source: BMC Bioinformatics. 2014 May 15;15:141. doi: 10.1186/1471-2105-15-141 (PMC4046028; doi:10.1186/1471-2105-15-141)
Supplement: Additional file 2 — DMRforPairs output for the comparison of A431-MCF7 and NA17018-NA17105. Please start from the HTML files in each folder. Available via the BMC Bioinformatics website. [file 1471-2105-15-141-S2.zip › 1394847754114233_MOESM2_ESM/A431_MCF7/figures/820.pdf]

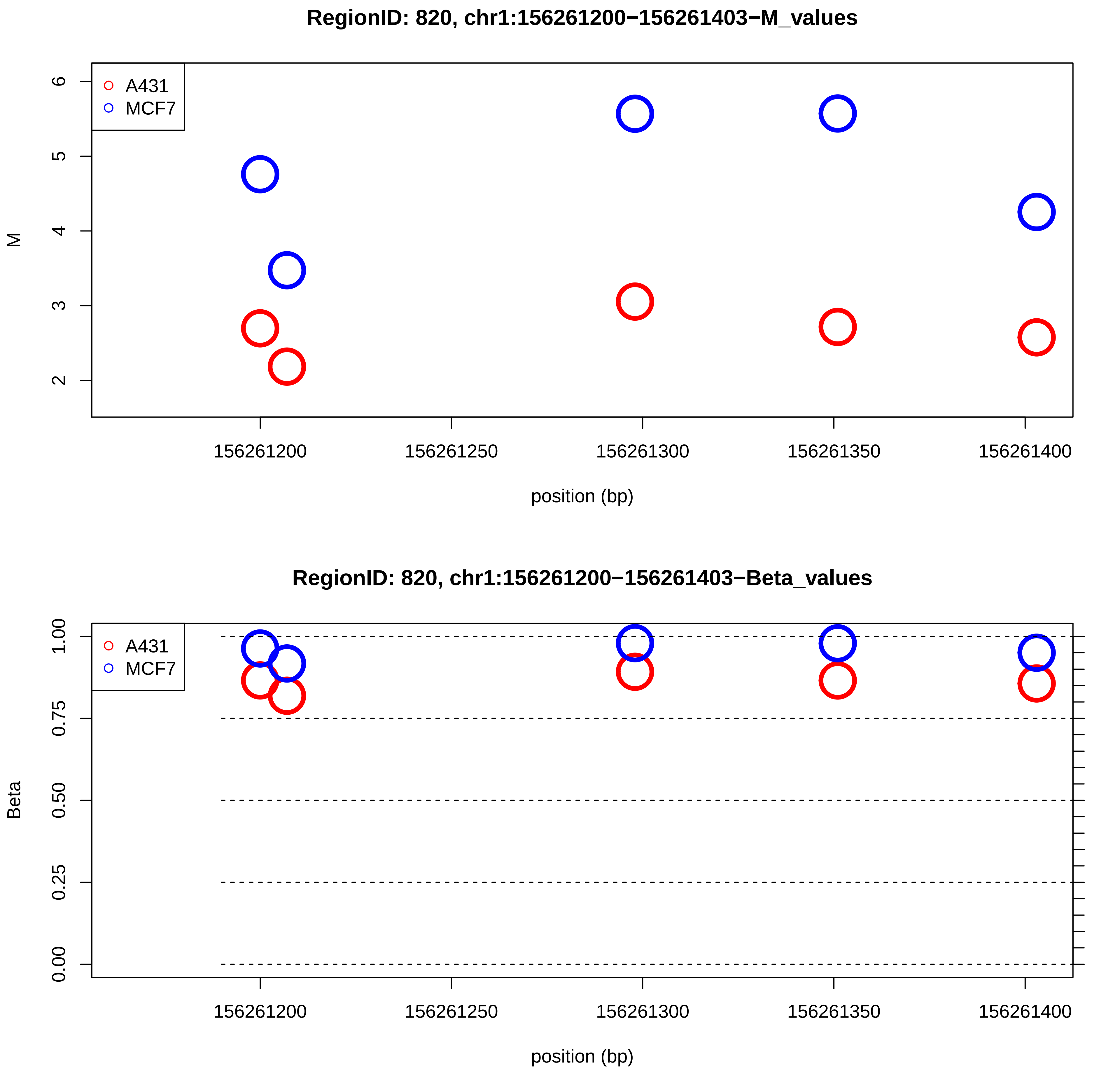

RegionID: 820, chr1:156261200−156261403−M_values
A431
MCF7
M
6
5
4
3
2
156261200
156261250
156261300
156261350
156261400
position (bp)
RegionID: 820, chr1:156261200−156261403−Beta_values
A431
MCF7
Beta
1.00
0.75
0.50
0.25
0.00
156261200
156261250
156261300
156261350
156261400
position (bp)

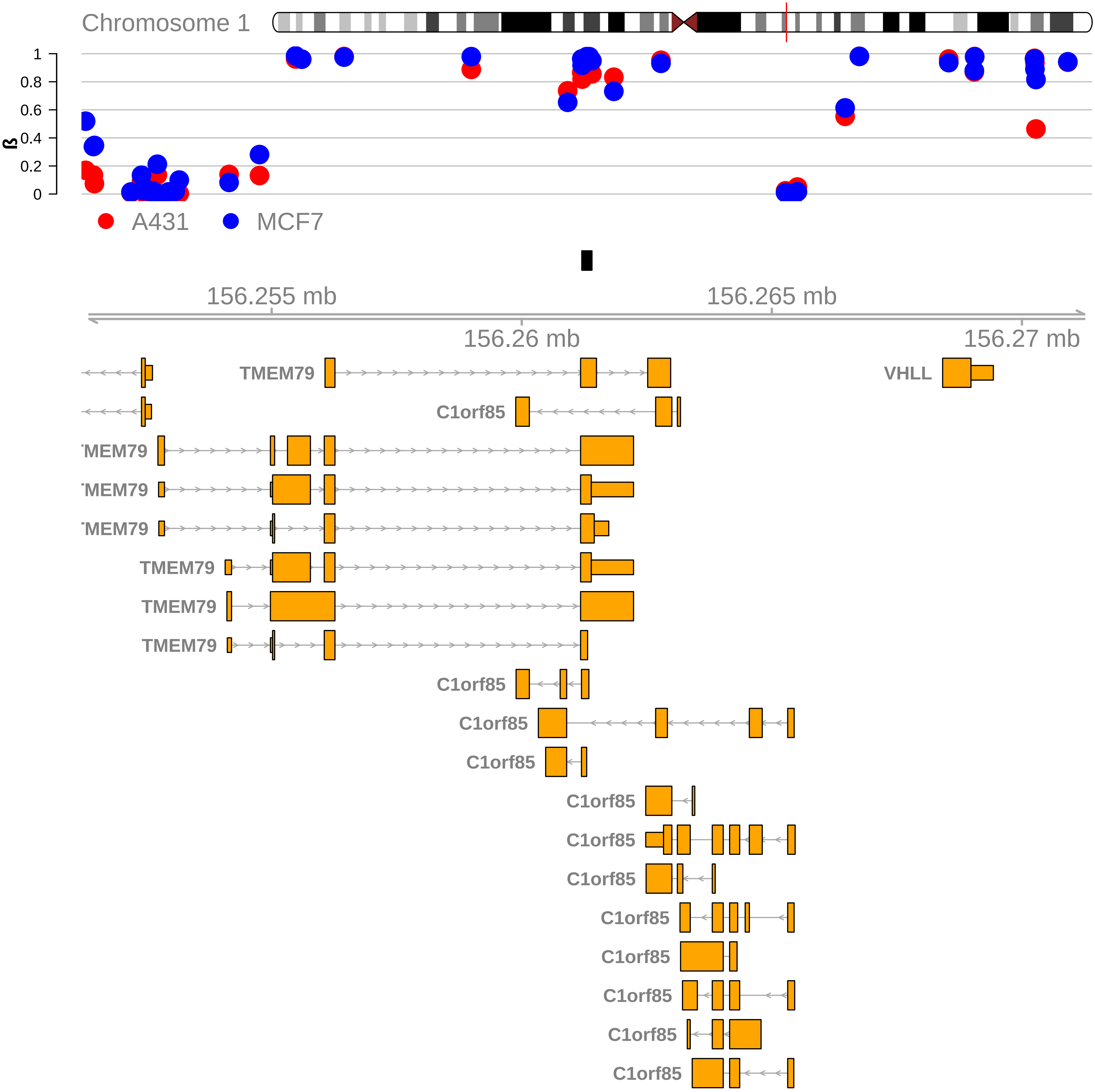

Chromosome 1
1
0.8
0.6
0.4
0.2
0
ß
A431
MCF7
156.255 mb
156.265 mb
156.26 mb
156.27 mb
TMEM79
VHLL
C1orf85
TMEM79
TMEM79
TMEM79
C1orf85
C1orf85
C1orf85
C1orf85
C1orf85
C1orf85
C1orf85
C1orf85
C1orf85
C1orf85
C1orf85
